# Risk factors for HIV virological non‐suppression among adolescents with common mental disorder symptoms in Zimbabwe: a cross‐sectional study

**DOI:** 10.1002/jia2.25773

**Published:** 2021-08-16

**Authors:** Victoria Simms, Sarah Bernays, Dixon Chibanda, Silindweyinkosi Chinoda, Abigail Mutsinze, Rhulani Beji‐Chauke, Owen Mugurungi, Tsitsi Apollo, Dorcas Sithole, Ruth Verhey, Helen A Weiss, Nicola Willis

**Affiliations:** ^1^ MRC International Statistics and Epidemiology Group London School of Hygiene & Tropical Medicine London UK; ^2^ London School of Hygiene & Tropical Medicine London UK; ^3^ Friendship Bench Harare Zimbabwe; ^4^ Department of Psychiatry University of Zimbabwe College of Health Sciences Harare Zimbabwe; ^5^ Africaid Harare Zimbabwe; ^6^ AIDS & TB Unit Ministry of Health and Child Care Harare Zimbabwe; ^7^ Mental Health Services Ministry of Health and Child Care Harare Zimbabwe

**Keywords:** adherence, adolescents, Africa, gender, social support, viral suppression

## Abstract

**Introduction:**

Adolescents are at increased risk of HIV virological non‐suppression compared to adults and younger children. Common mental disorders such as anxiety and depression are a barrier to adherence and virological suppression. The aim of this study was to identify factors associated with virological non‐suppression among adolescents living with HIV (ALWH) in Zimbabwe who had symptoms of common mental disorders.

**Methods:**

We utilized baseline data from a cluster‐randomized controlled trial of a problem‐solving therapy intervention to improve mental health and HIV viral suppression of ALWH. Sixty clinics within 10 districts were randomized 1:1 to either the intervention or control arm, with the aim to recruit 14 adolescents aged 10 to 19 per clinic. Adolescents were eligible if they scored ≥7 on the Shona Symptom Questionnaire measuring symptoms of common mental disorders. Multivariable mixed‐effects logistic regression was used to estimate odds ratios (OR) and 95% confidence intervals (95% CI) for factors associated with non‐suppression, defined as viral load ≥1000 copies/mL.

**Results:**

Between 2 January and 21 March 2019 the trial enrolled 842 participants aged 10 to 19 years (55.5% female, 58.8% aged <16). Most participants (N = 613) were taking an NNRTI‐based ART regimen (13 PI‐based, 216 unknown) and median duration on ART was six years (IQR three to nine years, 240 unknown). Of the 833 with viral load data 292 (35.1%) were non‐suppressed. Virological non‐suppression was independently associated with male sex (adjusted OR (aOR) = 1.43, 95% CI 1.04 to 1.97), and with not knowing one’s own HIV status (aOR = 1.77, 95% CI 1.08 to 2.88), or knowing one’s status but not disclosing it to anyone (aOR = 1.99, 95% CI 1.36 to 2.93), compared to adolescents who knew their status and had disclosed it to someone.

**Conclusions:**

ALWH with symptoms of common mental disorders have high prevalence of virological non‐suppression in Zimbabwe, especially if they do not know their status or have not disclosed it. In general adolescents should be informed of their HIV status, with encouragement on the beneficial health and social effects of viral suppression, to incentivise adherence. Efforts to strengthen the operationalization of disclosure guidelines for adolescents should now be prioritized.

## 1 Introduction

Adolescents (defined as aged 10 to 19 years), are the only age group in which, globally, HIV mortality is not falling [1]. Adolescents on antiretroviral therapy (ART) appear to be at higher risk of virological non‐suppression than adults, although evidence is limited. A systematic review in 2016 found only 20 papers reporting prevalence of virological suppression in adolescents aged 10 to 19 [2], and only seven papers from eastern and southern Africa where 70% of adolescents living with HIV (ALWH) reside [3]. A review of clinic records of 5715 adolescents aged 10 to 19 in Kenya found 33% were virologically non‐suppressed (viral load ≥1000 copies/µl) [4]. A baseline survey from a cluster‐randomized trial in Zimbabwe found that 46.8% of 496 adolescents on ART aged 13 to 19 were non‐suppressed at baseline [5], compared to 14.7% among adults in a national survey [6].

ALWH face numerous challenges to virological suppression. Adolescents with perinatally acquired HIV may have been exposed to monotherapy for prevention of mother to child transmission, followed by long‐term ART. Young adolescents are also vulnerable to over‐ or under‐treating due to prescription errors in weight‐based regimens. For example, a study of 309 children aged 0 to 17 years in Zimbabwe on weight‐based ART regimens found 36% were prescribed an incorrect dose [7]. Adolescents face additional challenges to ART adherence [8]. In a period of rapid cognitive and emotional development, emerging autonomy and limited resources [9], adolescents rely on family and social support to adhere to ART. They may be unaware or have limited understanding of their HIV status, reducing their motivation or capacity to adhere [10]. A study of 385 children aged six to fifteen in Zimbabwe found that 47.5% (n = 183) were unaware of their HIV status and the most common reasons caregivers gave for secrecy were that the child was too young (62%), would not understand the implications of an HIV diagnosis (56%), or might disclose it to others (26%) [11]. Adolescents may also conceal their treatment taking from others, which can practically impede their capacity to adhere [12]. ALWH have high rates of depression, anxiety and other common mental disorders (CMDs) [10], which inhibit adherence. A cross‐sectional study of 562 adolescents aged 12 to 18 in Malawi found 18.9% prevalence of depression using the Beck Depression Inventory II [13]. There is evidence ALWH are at higher risk of CMDs than their HIV‐unaffected peers, for example from a case‐control study in Rwanda that found 21% prevalence of suicidal behaviour among 218 ALWH aged 10 to 17 compared to 13% among neighbours unaffected by HIV, using the Youth Self‐Report Internalizing Subscale [14]. Parent‐reported symptoms of CMDs on the Pediatric Symptom Checklist were associated with virological non‐suppression in a cross‐sectional study of 692 children aged 8 to 16 in Botswana, although causality could not be determined [15]. Better understanding of risk factors for virological non‐suppression is needed in order to identify barriers and improve care outcomes. The aim of this study was to measure the prevalence of and identify risk factors for virological non‐suppression among ALWH with CMD symptoms in Zimbabwe.

## 2 Methods

The data for this paper are from the baseline survey of a cluster‐randomized controlled trial of problem‐solving therapy for adolescents which has been described in detail [16]. We present the descriptive characteristics of trial participants and factors associated with unsuppressed viral load, defined as ≥1000 copies/mL.

The trial was based in the World Health Organization (WHO) “best practice” [17] Zvandiri programme of trained, mentored young people known as community adolescent treatment supporters (CATS). We undertook a cluster‐randomized trial of the CATS intervention with 60 clinics randomized 1:1 within 10 districts to either the intervention or control arm. Control arm participants received standard of care from the CATS. In the intervention arm, CATS were trained in an adaptation of Friendship Bench problem‐solving therapy. The eligibility criteria were as follows: ALWH aged 10‐ to 19‐years old, on or initiating ART, who scored ≥7/14 on the Shona Symptom Questionnaire (SSQ‐14) of CMD symptoms [18]. Adolescents were excluded if they lived outside the area, were unable to comprehend the nature of the study (in either English, Shona or Ndebele), were in psychiatric care, or had end‐stage AIDS, psychosis, intoxication or dementia. Potential participants (adolescents either already registered with Zvandiri or who were patients at the clinic) were pre‐screened for eligibility and then invited to a trial orientation meeting. Those who were interested were screened for CMD symptoms by a research assistant. Enrolment ended when 14 participants had been enrolled. Participants aged 18 to 19 gave written informed consent. Those aged 10 to 17 gave written assent, and written informed consent was given by their caregiver.

A questionnaire was administered which included demographic, family and clinical characteristics, SSQ‐14 to measure symptoms of CMDs, the Patient Health Questionnaire (PHQ‐9) to measure symptoms of depression, and the WHO Disability Assessment Schedule (WHODAS 2.0) to measure difficulties caused by health conditions. The SSQ‐14 scores 1 point for each of 14 symptoms experienced within the previous two weeks [18]. Validated against the MINI‐KID in Zimbabwean ALHIV, the SSQ‐14 has a cutpoint of ≥8 (unpublished data). The PHQ‐9 consists of 9‐items measuring depression symptoms, each scored 0 to 3 on a Likert scale, with the total score (range 0 to 27) categorized into five groups [19]. It has been validated among adolescents in Chile [20]. The WHODAS 2.0 consists of 36 questions covering six domains (cognition, mobility, self‐care, getting along, life activities, social participation) [21]. It has been validated among adolescents in China [22]. Following that study, the question on sexual activities was dropped as it was not appropriate for the whole age group.

Adolescents with signs of psychological distress (visual/auditory hallucinations or suicidal ideation on the SSQ‐14, or ≥21 on the PHQ‐9) were referred to the clinic for further assessment and management, including follow‐up by a trained mental health nurse where available.

It was intended that data collection would be electronic, using an Open Data Kit (ODK) programme loaded onto tablets [16]. During recruitment a fault occurred which prevented the data management team from accessing uploaded data. From then on the study used the back‐up option of paper‐based data collection. Initially the processes of recruitment, consent procedures and the enrolment questionnaire were carried out by research assistants. After the switch to paper‐based data collection they were conducted by the CATS. Separate questionnaires were printed for participants who knew their own status and those who did not, with the “knows HIV status” version including a small number of extra questions such as ART regimen and date of HIV diagnosis. The “does not know status” version instead had questions such as whether the participant was taking any medication. The interviewer decided which questionnaire to use, based on whether the participant knew their own status, as logged in clinic records and reported by the caregiver. Double entry and validation of paper records was completed by a private company, Datalyst. The questionnaires are available for download (doi.org/10.17037/data.00002142).

A dried blood spot (DBS) fingerprick blood sample was collected from all participants and analysed at the National Microbiology Reference Laboratory to determine viral load. The minimum detection level was 840 copies/mL. Viral load results were returned to the clinics to aid in clinical management. Guidelines in Zimbabwe call for routine viral load testing to be carried out at 6 and 12 months after ART initiation, and then annually [23]. In case of virological failure (viral load ≥1000 copies), patients are given enhanced adherence counselling, a repeat viral load test after three months, and a switch to second line therapy if viral load remains high. Transition to third line therapy requires genotypic resistance testing, which is only available at one clinic.

Data were analysed using Stata v15. The WHODAS domain scores were recoded into “no problem” (score of 0) and “any problem” (score ≥1). A report of visual/auditory hallucinations or suicidal ideation on the SSQ‐14 was designated a “red flag.” The PHQ‐9 “moderately severe” (score 15 to 19) and “severe” (20 to 27) categories were combined due to small numbers. Univariable mixed‐effects logistic regression was used to identify variables associated with virological non‐suppression, with a random effect to allow for clinic‐level clustering. Factors associated with the outcome at significance level >10% were carried forward into a multivariable model, with age and sex retained *a priori*. Variables that were no longer associated with the outcome in multivariate analysis (*p* > 0.1) were removed. Knowledge of HIV status was defined as a participant who either (i) was pre‐determined by CATS to know their HIV status, based on clinic notes or (ii) when asked why they were taking medication, responded “for HIV.” Disclosure of status was defined as a participant who answered yes to the question “have you disclosed your HIV status to anyone” on the “knows HIV status” version of the questionnaire. Knowledge of status and disclosure to others were combined into a single categorical variable for the multivariable analysis to avoid collinearity. EQ5D scores were converted into index values [24] using quality of life state weightings developed in Zimbabwe in 2003 [25]. The association between age group and HIV status knowledge was estimated using odds ratio.

The trial was registered with the Pan African Clinical Trials Registry (PACTR201810756862405), and approved by the ethics committees of the Medical Research Council of Zimbabwe and the London School of Hygiene & Tropical Medicine.

## 3 Results

**Figure 1:**
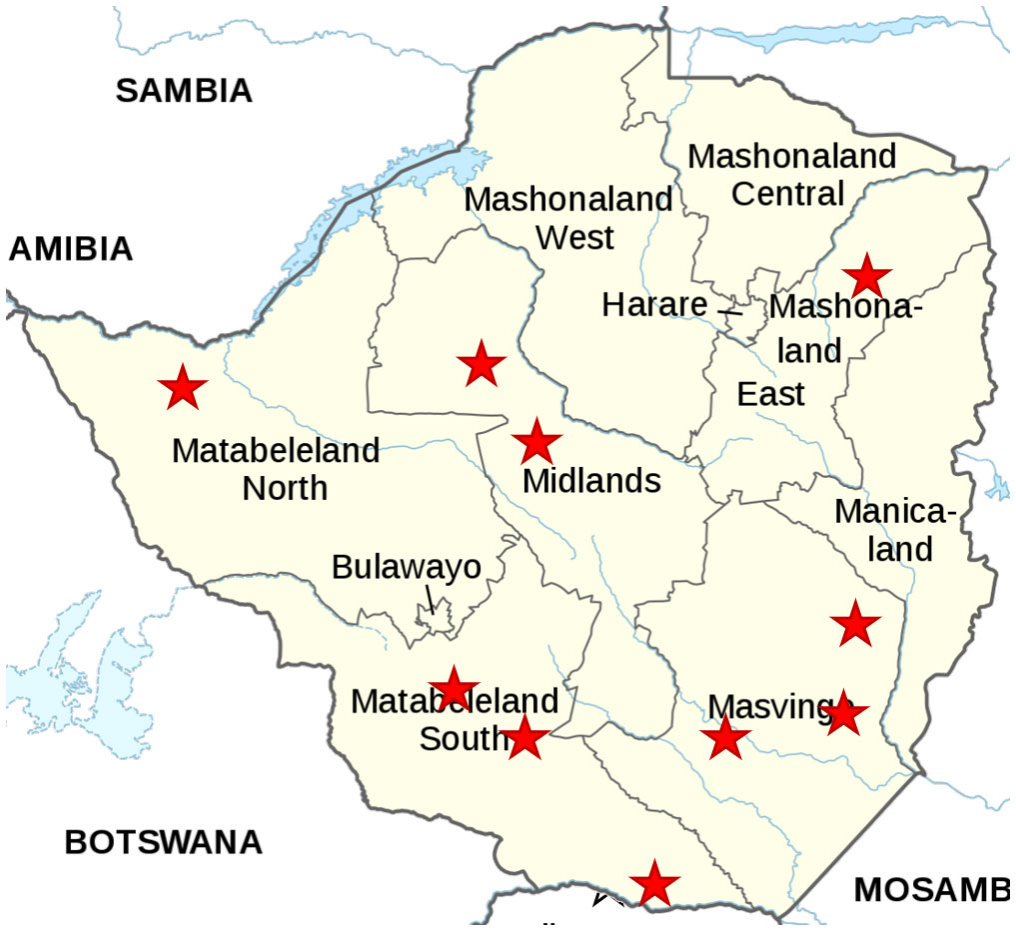
Map of trial sites.

Data collection took place between 2 January and 21 March 2019. The trial enrolled 842 participants (55.5% female), 84 per district in nine districts and 86 in the tenth (Figure 1). The first 232 (27.6%) questionnaires were completed electronically by a research assistant and the remaining 610 (72.4%) on paper by the CATS. The mean number of participants per clinic was 14.0 (standard deviation 2.7), range 6 to 22. Nine participants (1.1%) had no viral load test result. Of these, one participant died and two withdrew between enrolment and collection of a DBS sample, and for the remaining 6, samples were collected but not analysed.

Half (49.8%) of participants were single or double orphans (Table 1), and 474 (56.6%) participants’ primary caregiver was not a parent. Among adolescents aged ≥16 years, 90.1% had finished primary school and 3.9% were employed. Prevalence of self‐reported alcohol use and smoking increased with age, and was 5.1% and 4.4%, respectively, in 16‐ to 19‐year olds.

**Table 1:** Descriptive characteristics of trial participants by age group

	10 to 15 years, n (%)	16 to 19 years, n (%)	Total, n (%)
N	495 (58.8%)	347 (41.1%)	842 (100%)
Viral load (n = 833)			
<1000	314 (64.1)	227 (66.2)	541 (65.0)
1000 to 9999	114 (23.3)	59 (17.2)	173 (20.8)
10,000 to 99,999	50 (10.2)	50 (14.6)	100 (12.0)
≥100,000	12 (2.5)	7 (2.0)	19 (2.3)
Sex			
Female	270 (54.6)	197 (56.8)	467 (55.5)
Caregiver (N = 837)			
Mother	144 (29.3)	85 (24.6)	229 (27.4)
Father	34 (6.9)	17 (4.9)	51 (6.1)
Both parents	49 (10.0)	34 (9.8)	83 (9.9)
Grandparent	179 (36.5)	109 (31.5)	288 (34.4)
Aunt	43 (8.8)	45 (13.0)	88 (10.5)
Other	42 (8.6)	56 (16.2)	98 (11.7)
How often changed household (N = 839)			
Never	283 (57.3)	159 (46.1)	442 (52.7)
Once	119 (24.1)	98 (28.4)	217 (25.9)
Twice	61 (12.4)	56 (16.2)	117 (14.0)
3 times	18 (3.6)	14 (4.1)	32 (3.8)
4 times	5 (1.0)	8 (2.3)	13 (1.6)
5 or more times	8 (1.6)	10 (3.9)	18 (2.2)
Education level (N = 835)			
Below grade 7 (primary)	267 (54.4)	34 (9.9)	301 (36.1)
Grade 7 (end of primary)	166 (33.8)	120 (34.9)	286 (34.3)
O level (secondary)	58 (11.8)	182 (52.9)	240 (28.7)
A level (upper secondary)/tertiary	0	8 (2.3)	8 (1.0)
Orphan (N = 840)			
Both parents alive	256 (51.9)	166 (47.8)	422 (50.2)
One parent died	146 (29.6)	99 (28.5)	245 (29.2)
Both parents died	91 (18.5)	82 (23.6)	173 (20.6)
Knows their own HIV status			
Yes	364 (73.5)	320 (92.2)	684 (81.2)
Disclosed HIV status to anyone (N = 630/684)			
Yes	124 (38.2)	133 (43.6)	257 (40.8)
Drink alcohol (N = 814)			
Yes	7 (1.5)	17 (5.1)	24 (3.0)
Smoke (N = 828)			
Yes	5 (1.0)	15 (4.4)	20 (2.4)
Employed (N = 813)			
Yes	4 (0.8)	13 (3.9)	17 (2.1)
HIV transmission (N = 653)			
Vertical	190 (48.4)	163 (62.7)	353 (54.1)
Sexual/other	3 (0.8)	15 (5.8)	18 (2.8)
Don’t know	69 (17.6)	55 (21.2)	124 (19.0)
Not disclosed to	131 (33.3)	27 (10.4)	158 (24.2)
SSQ‐14 score			
Median (IQR)	8 (7 to 9)	8 (7 to 9)	8 (7 to 9)
SSQ‐14 red flag			
Yes	142 (28.7)	116 (33.4)	258 (30.6)
EQ5D index			
Median (IQR)	0.83 (0.74 to 1)	0.83 (0.74 to 1)	0.83 (0.74 to 1)
Depression symptoms (PHQ‐9)			
Minimal (0 to 4)	104 (21.1)	59 (17.0)	163 (19.4)
Mild (5 to 9)	216 (43.7)	166 (47.8)	382 (45.4)
Moderate (10 to 14)	130 (26.3)	92 (26.5)	222 (26.4)
Moderately severe/severe (15 to 27)	44 (8.9)	30 (8.7)	74 (8.8)
WHODAS			
Cognition			
Any difficulty	455 (92.1)	318 (91.6)	773 (91.9)
Mobility			
Any difficulty	363 (73.5)	247 (71.4)	610 (72.6)
Self‐care			
Any difficulty	326 (66.0)	192 (55.5)	518 (61.7)
Life activities			
Any difficulty	390 (79.0)	256 (73.8)	646 (76.8)
Participation			
Any difficulty	437 (88.5)	305 (88.2)	742 (88.3)
ART regimen and anchor drug			
First line (NNRTI)	322 (97.3)	291 (98.6)	613 (97.9)
TDF	259 (77.1)	277 (92.3)	536 (84.3)
AZT	56 (6.7)	13 (4.3)	69 (10.8)
ABC	7 (2.1)	1 (3.3)	8 (1.3)
Second line (PI)	9 (2.7)	4 (1.4)	13 (2.1)
LPV/r	3 (0.9)	2 (0.7)	5 (0.8)
ATV	5 (1.5)	2 (0.7)	7 (1.1)
Unknown	1 (0.3)	0	1 (0.2)
Unknown	164	52	216
Duration on ART (years)			
0	4 (1.3)	9 (3.1)	13 (2.2)
1 to 2	52 (16.9)	51 (17.3)	103 (17.1)
3 to 5	91 (29.6)	69 (23.4)	160 (26.6)
6 to 18	160 (52.1)	166 (56.3)	326 (54.2)
Unknown	188	52	240

Overall 35.2% of participants (n = 296) had symptoms of moderate, moderately severe or severe depression according to the PHQ‐9. The most common problems according to the WHODAS were cognitive (91.9%), followed by social participation (88.3%), life activities (76.8%), mobility (72.6%) and self‐care (61.7%).

Of 209 participants who completed the “does not know HIV status” questionnaire, 41 (19.6%) said they were taking medication for HIV and 10 (4.8%) said they were taking ART. A further 121 (57.9%) said they were taking medication but said it was for another reason, they did not know why, or did not give a reason. In Tables 1 and 2, the definition “knows HIV status” consisted of the 633 participants who completed a “knows status questionnaire” plus the 51 participants who completed a “does not know status” questionnaire but said unprompted that they were taking HIV medication or ART. ART regimen was known for 626 participants of whom 613 (97.9%) were taking an NNRTI‐based regimen. For 602 participants whose year of ART initiation was recorded, median length on ART was six years (IQR 3 to 9 years, range 0 to 18).

**Table 2:** Association of factors with virological non‐suppression

	N	Viral load ≥ 1000 c/mL, n (%)	Crude OR (95% CI)	*p*‐value	Adjusted OR (95% CI)	*p*‐value
N	833	292				
Sex						
Male	369	148 (40.1)	1.48 (1.10, 2.00)	0.01	1.43 (1.04, 1.97)	0.03
Female	464	144 (31.0)	1			
Age (years)						
10 to 12	230	78 (33.9)	1	0.50	1	0.40
13 to 15	260	98 (37.7)	1.18 (0.80, 1.74)		1.27 (0.83, 1.93)	
16 to 17	176	55 (31.2)	0.87 (0.56, 1.35)		0.93 (0.58, 1.51)	
18 to 19	167	61 (36.5)	1.13 (0.73, 1.75)		1.37 (0.84, 2.24)	
Primary caregiver (N = 828)						
Mother	225	81 (36.0)	1.27 (0.72, 2.25)	0.60		
Father	51	21 (41.2)	1.47 (0.68, 3.18)			
Both parents	82	24 (29.3)	1			
Grandparent	287	99 (34.5)	1.24 (0.71, 2.17)			
Aunt	86	36 (41.9)	1.59 (0.82, 3.10)			
Other	97	29 (29.9)	0.95 (0.48, 1.85)			
Religion (N = 822)						
Catholic	91	36 (39.6)	1.57 (0.89, 2.77)	0.46		
Adventist	83	30 (36.1)	1.30 (0.73, 2.34)			
Methodist	62	22 (35.4)	1.30 (0.69, 2.48)			
Pentecostal	267	89 (33.3)	1.08 (0.69, 1.69)			
Apostolic	77	26 (33.8)	1.15 (0.61, 2.17)			
ZCC	68	31 (45.6)	1.95 (1.05, 3.62)			
None	24	9 (37.5)	1.20 (0.44, 3.26)			
Other	150	46 (30.7)	1			
Ever changed household (N = 830)						
No	437	146 (33.4)	1			
Yes	393	144 (36.9)	1.19 (0.88, 1.61)	0.26		
Education						
Below grade 7	297	110 (37.0)	1	0.33		
Grade 7	284	103 (36.3)	0.94 (0.66, 1.34)			
Secondary/Tertiary	245	76 (31.0)	0.75 (0.51, 1.11)			
Orphan						
No	417	141 (33.8)	1	0.58		
One parent died	242	84 (34.7)	1.01 (0.71, 1.44)			
Both parents died	172	66 (38.4)	1.22 (0.83, 1.79)			
Knowledge/disclosure of HIV status						
Knows status, disclosed	254	65 (25.6)	1		1	
Knows status, has not disclosed to others	367	143 (39.0)	1.91 (1.31, 2.79)	0.001	1.99 (1.36, 2.93)	<0.001
Does not know status	158	60 (38.0)	1.66 (1.05, 2.64)	0.031	1.77 (1.08, 2.88)	0.02
Drink alcohol (N = 806)						
No	782	279 (35.7)				
Yes	24	7 (29.2)	0.77 (0.30, 1.95)	0.58		
HIV transmission						
Vertical	348	111 (31.9)	1	0.20		
Sexual	17	3 (17.7)	0.45 (0.12, 1.65)			
Don’t know	123	50 (40.7)	1.43 (0.91, 2.26)			
Other/unaware	335	124 (37.0)	1.21 (0.86, 1.71)			
Depression symptoms (PHQ‐9 category)						
Minimal (0 to 4)	161	56 (34.8)	1	0.48		
Mild (5 to 9)	378	126 (33.3)	0.90 (0.60, 1.36)			
Moderate (10 to 14)	219	79 (36.1)	1.07 (0.67, 1.70)			
Moderately severe/severe (15 to 27)	74	30 (40.5)	1.38 (0.74, 2.54)			
SSQ‐14						
Red flag	256	96 (37.5)	1.14 (0.82, 1.59)	0.42		
No red flag	577	196 (34.0)	1			
EQ5D						
No difficulty	280	93 (33.2)	1	0.15		
Any difficulty (any score > 1)	435	148 (34.0)	1.00 (0.71, 1.40)			
Severe difficulty (total score > 8)	116	50 (43.1)	1.53 (0.95, 2.48)			
WHODAS cognition						
Any difficulty	764	262 (34.3)	0.58 (0.33, 1.02)	0.06		
No difficulty	68	29 (42.7)	1			
WHODAS mobility						
Any difficulty	603	204 (33.8)	0.83 (0.59, 1.17)	0.30		
No difficulty	228	85 (38.2)	1			
WHODAS self‐care						
Any difficulty	510	179 (35.1)	1.04 (0.76, 1.43)	0.81		
No difficulty	3121	112 (34.9)	1			
WHODAS life activities						
Any difficulty	638	209 (32.8)	0.65 (0.45, 0.92)	0.02		
No difficulty	194	82 (42.3)	1			
WHODAS participation						
Any difficulty	733	247 (33.7)	0.59 (0.37, 0.94)	0.03		
No difficulty	98	44 (44.9)	1			
ART regimen						
TDF (first line)	536	173 (32.5)	0.73 (0.51, 1.04)	0.61		
AZT (first line)	69	25 (37.3)	0.89 (0.49, 1.63)			
ABC (first line)	8	3 (42.9)	1.16 (0.24, 5.58)			
LPV/r (second line)	5	1 (25.0)	0.48 (0.05, 4.97)			
ATV (second line)	7	2 (28.6)	0.64 (0.12, 3.61)			
Unknown (second line)	1	1 (100.0)	–			
Unknown	216	87 (40.5)	1			
Duration on ART (years) (N = 602)						
0	13	3 (23.1)	0.45 (0.12, 1.75)	0.18		
1 to 2	102	31 (30.4)	0.75 (0.45, 1.25)			
3 to 5	159	45 (28.3)	0.65 (0.42, 1.01)			
6 to 18	319	120 (37.6)	1			

The proportion of adolescents who knew they were HIV positive increased with age from 73.5% in 10‐ to 15‐year olds (the reference group) to 92.2% in 16‐ to 19‐year olds (OR = 4.83, 95% CI 2.98 to 7.82; *p* < 0.001). Most (59.2%) adolescents who knew their own status had not told anyone else, although the proportion who had disclosed was higher in the 16 to 19 group (OR = 1.47, 95% CI 1.01 to 2.14; *p* = 0.045).

Of the 257 adolescents who had disclosed their status, 251 reported to whom they had disclosed. The majority (197, 78.5%) had disclosed to a relative, 23 (9.2%) to a friend, 12 (4.8%) to a boyfriend/girlfriend and 21 (7.0%) to someone else. Only 41 participants reported a reason for disclosing their status, with the most common reasons being to receive care and support (36.6%) or adherence support specifically (29.3%).

Among the 833 participants with a viral load test result, 541 (64.9%) had a viral load <1000 copies/mL, 173 (20.8%) 1000 to 9999 copies/mL, 100 (12.0%) 10,000 to 99,999 copies/mL and 19 (2.3%) ≥100,000 copies/mL, with a maximum of 788,592. The prevalence of virological non‐suppression was 35.1% (n = 292), ranging by district from 20.2% to 55.4%.

**Figure 2:**
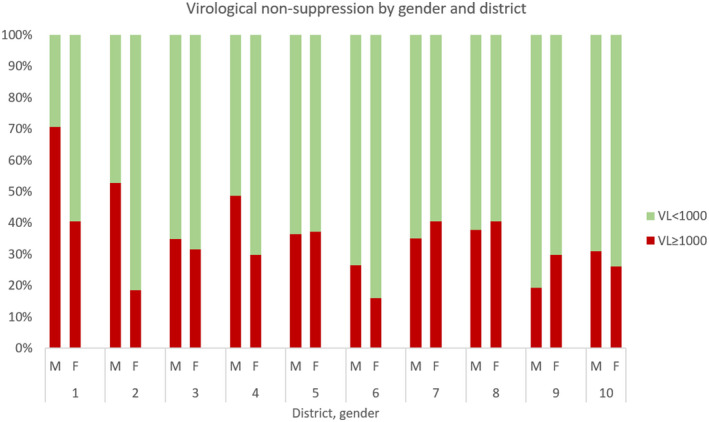
Prevalence of virological non‐suppression by gender and district.

Males had higher odds of virological non‐suppression than females, (40.1% vs. 31.0%, OR = 1.43, 95% CI 1.04, 1.97; *p* = 0.03) (Table 2). Figure 2 presents the prevalence of virological non‐suppression by sex and district. There was no evidence that virological non‐suppression was associated with age, education, primary carer or orphanhood, knowledge of HIV status as a binary variable, ART regimen or duration on ART (Table 2). Among participants who knew their HIV status, non‐disclosure was associated with non‐suppression (39.0% vs. 25.6%, OR = 1.99, 95% CI 1.36, 2.93; *p* < 0.001). In univariable analysis, there was no evidence of an association between depression symptoms score and virological non‐suppression (Table 2). Participants who reported difficulties with life activities, social participation or cognition were less likely to have virological non‐suppression.

In the final multivariable model (Table 2), virological non‐suppression was independently associated with being male (adjusted OR = 1.43, 95% CI 1.04, 1.97; *p* = 0.03), not knowing one’s own HIV status (aOR = 1.77, 95% CI 1.08, 2.88; *p* = 0.02) and not disclosing HIV status to someone else (aOR = 1.99, 95% CI 1.36, 2.93; *p* < 0.001), compared to those who knew their status and had disclosed it.

## 4 Discussion

Among these ALWH in Zimbabwe virological non‐suppression was very high (35.1%), demonstrating the challenges adolescents face in attaining viral suppression. This is comparable with the prevalence of virological non‐suppression among ALWH found in Kenya (33% of 5715) [4], and a previous study in Zimbabwe found even higher prevalence (46.8% of 496) [5]. Three key factors were associated with virological non‐suppression: male sex, not knowing one’s own HIV status and not disclosing HIV status.

Achieving viral suppression requires two essential elements: access to effective treatment and optimal adherence behaviour. Access to effective treatment requires patients to be able to get to the clinic, collect drug refills and be prescribed an ARV regimen to which they are not resistant. Zimbabwe has a “Treat All” approach whereby anyone with HIV is eligible for ART initiation regardless of clinical stage or CD4 count, and the preferred first line regimen at the time of data collection was TDF+3TC+EFV [23]. Resistance testing is sporadic and is not yet a component of standard of care in Zimbabwe. A WHO report in 2019 found pre‐treatment drug resistance to NNRTIs (efavirenz and nevirapine) in Zimbabwe of 11% among 353 adult first‐line ART initiators and over 60% among 227 newly diagnosed infants [26]. In 2016 in Harare, a voluntary organization‐funded clinic provided enhanced adherence counselling to 726 young people aged 16 to 24 on first‐line ART. After the counselling, 74 (10.2%) had confirmed virological failure and 72/74 (97%) had drug resistance mutations [27]. Out of 102 children and adolescents in Harare with virological failure in 2012, 67.6% had ≥1 clinically significant mutation [28]. There is increased emphasis on treatment switching in international guidelines [29], informed by a growing recognition of high resistance rates among ALWH [30]. In this study the highest prevalence of non‐suppression, by far, was in District 1, a border town with high HIV prevalence. It is likely that participants travelled across the border for work which may have hindered their access to ART. Reflecting the mobility of youth populations, it is vital that clinics are flexible in terms of supporting three‐month drug refills and weekend or after‐hours clinic visits [31, 32].

The second critical component in viral suppression is sustained adherence. It is likely that the effects of sex, HIV status knowledge and status disclosure on virological non‐suppression are caused by differential adherence behaviour. In our study, males were at increased risk of non‐suppression. The finding is consistent with the literature from adults living with HIV [33, 34], and is increasingly noted within adolescents too, in recent studies from Ethiopia [35], Malawi [36] and Kenya [4]. However, a systematic review of adolescent ART adherence in low and middle‐income countries (8/15 studies from sub‐Saharan Africa) found no consistent association with sex [8]. Healthcare is often perceived as “female” [37] and underutilized by men [38]. Among adolescents too, boys are less likely than girls to seek healthcare, possibly because boys are socialized to more highly value being perceived as self‐reliant [39]. In KwaZulu Natal, South Africa, only 20% of 15‐ to 19‐year olds who visited clinics were male [40]. Boys and men also have lower engagement in psychosocial support services, indicating a need for tailored approaches to suit them [38].

Participants who knew they were HIV positive had better odds of virological suppression, in line with evidence that adolescents who know their status have improved ART adherence [41, 42, 43]. However, a systematic review of the effect of disclosure on children’s adherence (aged 0 to 19) in resource‐limited settings found conflicting results [44]. One reason for the inconsistency could be that “knowing one’s own HIV status” can encompass a broad spectrum of stages of understanding what it means to be HIV positive [45], whereas most studies (including this one) reduce it to a binary variable. Adolescents who know they are living with HIV frequently have little idea what that means for them, apart from a moral imperative to take daily medication for life [46]. There is often little discussion of what it will mean to grow up and live with HIV, and after the revelation is made children are advised not to think about their diagnosis [11, 47]. In contrast, WHO guidance recommends that caregivers should incrementally extend children’s knowledge of HIV over time, in pace with their cognitive and emotional development [48]. Further, enhanced adherence counselling should be provided for all adolescents with high viral load, to identify and address adherence barriers and to prevent unnecessary regimen switching.

Caregivers of children with perinatally acquired HIV can be reticent to tell the child their HIV status. Their concerns relate to the impact they anticipate the knowledge would have on their child, and also to the complex, relational entanglement involved in the admission [49, 50]. Caregivers and healthcare staff can also underestimate adolescents’ understanding of living with HIV [51]. In this study, participants’ responses to questions about what medication they took and its purpose revealed that at least 51 adolescents knew they were HIV positive even though clinic records and caregivers said that they did not know. Similar results have been found in South Africa [52] and Uganda [53]. A qualitative study in Uganda indicated that adolescents found their caregivers’ silence frustrating [49].

Viral suppression, achieved through optimal adherence, can support thriving health and can be socially/relationally enabling, by reducing physical signs of HIV and removing fears of onward transmission [5, 54]. Open discussion of the implications of an HIV status may help adolescents to “make sense” of their lived reality [55], enable them to develop a realistic, positive outlook for the future, and thereby have improved mental health and wellbeing [53]. National guidelines state that caregivers who find it difficult to disclose to the child should be supported by healthcare workers [23], and this aligns with evidence from systematic reviews [56, 57].

In this study, adolescents who knew their own HIV status but had not told anyone else were at almost twice the odds of virological non‐suppression after adjusting for covariates. Similarly, in South Africa, non‐disclosure was associated with lower ART adherence [52]. Adolescents’ experiences of HIV are typically characterized by isolation, stigma and shame, which impede their adherence [12, 58]. They are concerned to protect social relationships by concealing their HIV status and use of ART [59]. Many caregivers, and sometimes healthcare workers, actively discourage ALWH from disclosing their status, thereby cutting them off from potential sources of support [45]. Without access to formal HIV psychosocial support [60], this further exacerbates the cycle of loneliness and suboptimal adherence. In this study, the most common reason why adolescents disclosed their status was so they could access case and support, often specifically to help them adhere.

Study participants who had disclosed their status to another person reported more cognitive, life participation and self‐care problems on the WHODAS, indicating that disclosure can have drawbacks as well as benefits. This is reflected in the literature, where adolescents often feel ambivalent about disclosure, knowing it is inevitable at some point, but unsure how to recognize the right time or person [61]. They need support to make decisions about whom to tell and advice about how to tell [62]. Disclosure of HIV status may pose substantial risks for adolescents and damage social relationships [45], with subsequent negative effects on adherence and mental health [63]. In particular, the guidance to always disclose to partners can be very challenging and may be a barrier to remaining in care. Discussions of adherence could instead be focused around U = U and the protective effects of viral suppression [5].

The strengths of the study are that it consists of a large, representative sample of adolescents in HIV care from all over the country, including both urban and rural areas. CATS established a rapport with participants which helped them collect reliable self‐reported data. The tools have been validated in Zimbabwe, albeit in adult populations. The PHQ‐9 and WHODAS 2.0 have been validated among adolescents [20, 22]. A limitation of the study is that it was a trial population. However, the response rate among those who were eligible for enrolment was very high (97.6%), limiting selection bias. Unsuppressed viral load was not confirmed with a second test as recommended by the WHO. The study was cross‐sectional and temporal relationships cannot be established. The date of ART initiation was not recorded and some participants may have initiated ART within three months of enrolment, not allowing sufficient time for viral suppression. The unplanned switch from electronic to paper‐based data collection caused limitations. Data collection was done by the CATS rather than by independent research assistants, and this could have led to social desirability bias. Copies of paper questionnaires were of poor quality and inconsistent formatting, causing problems such as loss of item numbers, which made administration difficult. Some clinics ran short of questionnaires and may have used a “disclosed” questionnaire when a “non‐disclosed” one was called for, adding to the confusion around knowledge of status. The questionnaire was also long and burdensome for children, especially the 30‐item WHODAS which came last. As a result, WHODAS information was unreliable. At follow‐up data collection these problems were resolved with the use of robust electronic data collection systems.

## 5 Conclusions

ALWH with symptoms of CMDs have poor virological suppression in Zimbabwe. This could be improved by telling adolescents their HIV status to incentivize adherence behaviour, emphasizing the health effects of viral suppression and related positive social/relational impacts of adherence. Global and national guidelines recommend disclosure of HIV status to ALWH. Strengthened operationalization of these guidelines is needed to support adolescents’ mental health and incentivize adherence behaviour. Adolescents should also be counselled on the potential benefits and consequences of disclosing to others. Caregivers may benefit from advice on how to disclose to adolescents that they are HIV positive and how to make disclosure an ongoing, revisited conversation.

## Competing interest

The authors declare no conflict of interest.

## Authors’ contributions

DC, HAW and NW designed the study. SC, AM and RBC performed the research. RV and DC designed the intervention. VS analysed the data. OM, TA and DS contributed essential support. VS and SB wrote the paper. All authors have read and approved the final manuscript.
